# *Gemini*, a Bifunctional Enzymatic and Fluorescent Reporter of Gene Expression

**DOI:** 10.1371/journal.pone.0007569

**Published:** 2009-11-04

**Authors:** Lance Martin, Austin Che, Drew Endy

**Affiliations:** 1 Department of Bioengineering, Stanford University, Stanford, California, United States of America; 2 MIT Computer Science & Artificial Intelligence Laboratory, Cambridge, Massachusetts, United States of America; Center for Genomic Regulation, Spain

## Abstract

**Background:**

The development of collections of quantitatively characterized standard biological parts should facilitate the engineering of increasingly complex and novel biological systems. The existing enzymatic and fluorescent reporters that are used to characterize biological part functions exhibit strengths and limitations. Combining both enzymatic and fluorescence activities within a single reporter protein would provide a useful tool for biological part characterization.

**Methodology/Principal Findings:**

Here, we describe the construction and quantitative characterization of *Gemini*, a fusion between the β-galactosidase (β-gal) α-fragment and the N-terminus of full-length green fluorescent protein (GFP). We show that *Gemini* exhibits functional β-gal activity, which we assay with plates and fluorometry, and functional GFP activity, which we assay with fluorometry and microscopy. We show that the protein fusion increases the sensitivity of β-gal activity and decreases the sensitivity of GFP.

**Conclusions/Significance:**

*Gemini* is therefore a bifunctional reporter with a wider dynamic range than the β-gal α-fragment or GFP alone. *Gemini* enables the characterization of gene expression, screening assays via enzymatic activity, and quantitative single-cell microscopy or FACS via fluorescence activity. The analytical flexibility afforded by *Gemini* will likely increase the efficiency of research, particularly for screening and characterization of libraries of standard biological parts.

## Introduction

The number of standard biological parts available to researchers within the synthetic biology community has grown considerably in recent years. For example, the Registry of Standard Biological Parts has expanded from under 50 parts in 2003 to over 3000 parts by 2009 [1]. However, many existing parts have not been quantitatively characterized [2]. As a result, it is difficult to accurately predict the behavior of a biological system composed of such parts. Thus, current engineering practice typically requires months of testing and tuning before the hoped-for system functions as desired [3]–[4]. The inability to select parts based upon their characterized behavior, or to predict how parts will function together, may help to explain why the complexity of systems assembled by synthetic biologists may have reached a plateau [5]. If true, then developing improved collections of well-characterized standard biological parts would support the more efficient engineering of increasingly complex and novel biological systems.

Quantitative part characterization seems central to the engineering of biological systems for several reasons. In general, part characterization aids the development of mathematical models that help engineers to simulate the behavior of many component biological systems prior to physical testing [6]. More specifically, quantitative transfer functions that characterize the relationship between input and output signals help engineers to determine whether the output of one part is compatible, or might be made compatible, with the input of another [7]–[8]. Additionally, engineers can choose characterized parts based upon other quantitative criteria, such as material and energy resource loads on a host cell, thereby further reducing the chance that a system might fail to behave as expected.

Genetically encoded reporter proteins are important and widely used tools for quantification of promoter activity, translational efficiency, and protein localization, trafficking, and stability. Many standard biological parts utilize or depend upon these biochemical functions [5]. Thus, reporter proteins are widely used for screening the activity and quantifying the dynamic behavior of standard biological parts [9]–[10]. Going forward, the synthetic biology community would benefit from next generation reporters that improve upon at least four features common to most reporters. First, reporters should function in chassis commonly used by synthetic biologists. Second, reporters should exhibit wide dynamic range, meaning their activity should be quantifiable when they are either weakly or strongly expressed. Third, the dynamic range of reporters should be characterized across a range of commonly used expression levels, and should be correlated with measurement reference standards if available. Fourth, reporters should enable analytical flexibility, ranging from high-throughput screening to quantitative single cell assays.

The enzymatic and fluorescent reporter proteins currently used to characterize biological parts have strengths and limitations. For example, enzymatic reporters, such as β-galactosidase (β-gal), react with an externally supplied substrate and yield a detectable product. β-gal has been very effective for high-throughput screening assays, such as yeast two-hybrid protein interaction mapping [11], for at least two reasons. First, β-gal exhibits high sensitivity because even a small amount of enzyme can, over time, catalytically process many substrate molecules to produce a detectable signal. Second, colorimetric detection of β-gal activity with the naked eye using convenient and inexpensive plate assays is possible. However, the utility of β-gal is also limited. Some β-gal assays cannot be performed in live cells because substrate penetration is lethal [12]. Also, while suitable for population-average measurements, β-gal cannot be easily used for high-throughput quantification within single cells.

As a second example, fluorescent reporters, such as the green fluorescent protein (GFP), contain a chromophore intrinsic to the protein that emits a detectable fluorescent signal after excitation by light of specific wavelengths [13]–[14]. Because fluorescent reporters do not require an exogenous chemical substrate, they can be used for protein localization studies in live cells and for quantification of gene expression using time-lapse fluorescence microscopy. In addition, fluorimetry enables population-averaged measurements of expression strength, and fluorescence-activated cell sorting enables high-throughput quantification of expression strength across single cells. However, the utility of GFP is limited, in that each molecule provides only one chromophore that will photobleach over time. As a result, GFP is difficult to detect when weakly expressed, particularly in media or cells having high autofluorescence [14].

With their intrinsic strengths and limitations in mind, neither GFP nor β-gal might be individually best suited to meet the abovementioned needs of the synthetic biology community. While the parallel, independent assembly of test constructs using both GFP and β-gal is reasonable for some small-scale studies, such duplication of effort becomes impractical as the number of parts to be characterized increases [15]. However, bifunctional reporters that combined the enzymatic activity of β-gal and fluorescence activity of GFP, or equivalent, may be well suited for such work: bifunctional reporters may enable the detection of weak reporter expression while also supporting screening assays via enzymatic activity; at the same time, bifunctional reporters may enable time-lapse fluorescence microscopy or FACS, thereby supporting measurement of population heterogeneity via fluorescence; finally, the analytical flexibility afforded by bifunctional reporters might be useful for characterizing component libraries or many component sets of biological parts.

With these benefits in mind, we found two examples of bifunctional reporters that were previously developed for specific research projects. One bifunctional reporter, which is designed for use in CHO cells, loach, and chicken embryos, combines full length LacZ and GFP coding sequences that are optimized for expression in humans [16]. A second bifunctional reporter is a fusion between a *C. elegens* heat shock protein, GFP, and full-length LacZ [12]. In this work, we describe the development and quantitative characterization of an improved bifunctional reporter that is optimized for use in microbes, and that is designed to support the characterization of many standard biological parts.

## Results

We engineered *Gemini*, a fusion protein encoding both the enzymatic activity of β-gal and the fluorescence activity of GFP. We designed a fusion reporter, rather than a polycistronic reporter, because we hypothesized that the physical coupling of GFP and β-gal would allow engineers to make more comparable fluorescent and enzymatic measurements than with independent GFP and β-gal proteins. *Gemini* incorporates the β-gal α-fragment rather than the full-length LacZ coding sequence; the β-gal α-fragment is able to reconstitute functional β-gal activity via complementation with the β-gal omega-fragment, which must be pre-expressed within the cell [17]. We chose to use the β-gal α-fragment because it is encoded by only 180 base pairs, significantly less than the 3,075 base pair coding sequence of full-length LacZ. Due to its reduced coding sequence length, the α-fragment is easier to clone than full length LacZ. We assembled *Gemini* by fusing two BioBrick parts, the β-gal α-fragment (BBa_E0038) and GFP (BBa_E0043), resulting in a standardized bifunctional reporter that can be connected to other BioBrick parts via BioBrick assembly standard #10 [18].

### Bifunctional reporter design

**Figure 1:**
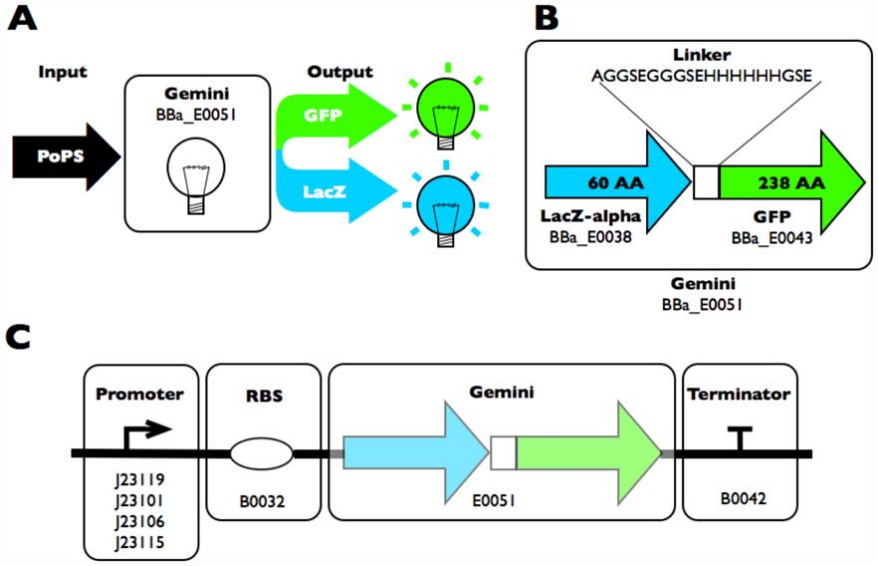
Function, design, and expected use of *Gemini*, a bifunctional reporter of gene expression. **A**) A PoPS input signal, the number of arriving polymerases per second, drives expression of *Gemini*. **B**) *Gemini* (BBa_E0051) is a fusion reporter composed of both full-length GFP (BBa_E0043) and the β-gal α-fragment (BBa_E0038). Full-length GFP is fused with a 19 amino acid linker containing a six Histidine tag and predominantly Glycine and Serine residues to the β-gal α-fragment. **C**) For characterization of its enzymatic and fluorescence activities across a range of input PoPS, we placed *Gemini* under the control of a medium strength RBS (BBa_B0032) and four different promoters of variable strength (in order of decreasing strength BBa_J23119, 101, 106, 115) on a low-copy vector (BBa_PSB4A5).

We built and tested two different bifunctional reporter designs (Figure S1). In the first design, the N-terminus of the β-gal α-fragment (BBa_E0038) is fused directly to the C-terminus of full-length GFP (BBa_E0043). In the second design, the N-terminus of full-length GFP is fused to the C-terminus of the β-gal α-fragment via a linker (Figure 1b). Initial experiments using the first design yielded functional enzymatic activity, but poor fluorescence activity (not shown). Initial experiments with the second design yielded marked fluorescence and enzymatic activity. We named the second design *Gemini* and studied it further.

### Gemini exhibits functional β-gal and GFP activity

We first confirmed that *Gemini* encodes functional fluorescence and enzymatic activities using microscopy and X-gal plates, respectively. We wanted to fairly compare the fluorescence and enzymatic activities of *Gemini* to the individual activities of GFP and the β-gal α-fragment alone across a range of commonly used expression levels. To do this, we placed the coding sequences for each of the three reporters under the control of the same expression cassettes and vector backbones. Specifically, we placed all three reporters under the control of a medium strength RBS (BBa_B0032) and four different promoters of variable strength (in order of decreasing strength BBa_J23119, 101, 106, 115) on a low-copy vector (BBa_PSB4A5).

Thus, we built four promoter variants for each reporter: *Gemini* (Figure 1C), the β-gal α-fragment alone (Figure S2a), and GFP alone (Figure S2b). The four promoters were chosen for two reasons. First, the four promoters are commonly used standard BioBrick parts. Second, the four promoters span a wide range of transcriptional activity (Methods).

**Figure 2:**
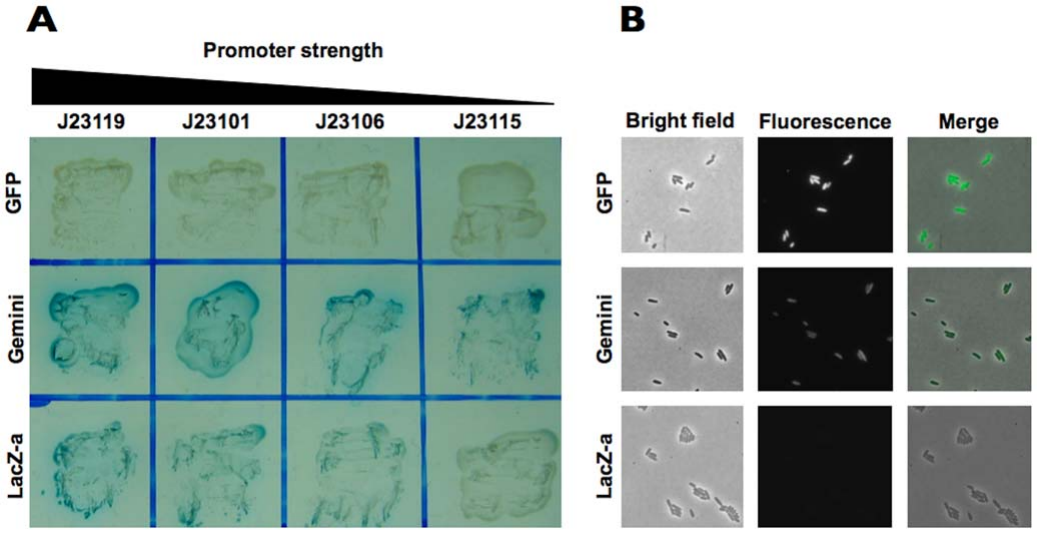
*Gemini* encodes both functional enzymatic and fluorescence activities. We wanted to fairly compare the fluorescence and enzymatic activities of *Gemini* to the individual activities of GFP and the β-gal α-fragment alone. To do this, we placed the coding sequences for GFP and the β-gal α-fragment under the control of the same expression cassettes as *Gemini*. Specifically, we placed all three reporters under the control of a medium strength RBS (BBa_B0032) and four different promoters of variable strength (in order of decreasing strength BBa_J23119, 101, 106, 115) on a low-copy vector (BBa_PSB4A5). Thus, we built four promoter variants for each reporter. **A**) To test enzymatic activity, we grew cells containing promoter variants for *Gemini*, GFP, and the β–gal α-fragment on a plate containing X-gal and IPTG. The plate-based experiments indicated that the enzymatic activity of *Gemini* is stronger than that of the β-gal α-fragment alone. **B**) To test fluorescence activity, we grew cells containing *Gemini*, GFP, and α-fragment expression constructs with promoter J23101 in supplemented M9 media. An aliquot was sampled in mid-exponential growth phase for microscopy measurements. Microscopy measurements indicated that the fluorescence activity of *Gemini* is functional and weaker than that of GFP alone.

To test enzymatic activity we grew cells containing promoter variants for *Gemini*, GFP, and the β–gal α-fragment on a plate containing X-gal and IPTG. The plate-based experiments indicated that the enzymatic activity of *Gemini* is stronger than that of β-gal α-fragment alone (Figure 2a). To test fluorescence activity, we grew cells containing *Gemini*, GFP, and α-fragment expression constructs with promoter J23101 in supplemented M9 media at 37°C. We sampled an aliquot in mid-exponential growth phase for microscopy measurements, which indicated that the fluorescence activity of *Gemini* is weaker than that of GFP alone (Figure 2b).

### Quantitative characterization of Gemini

**Figure 3:**
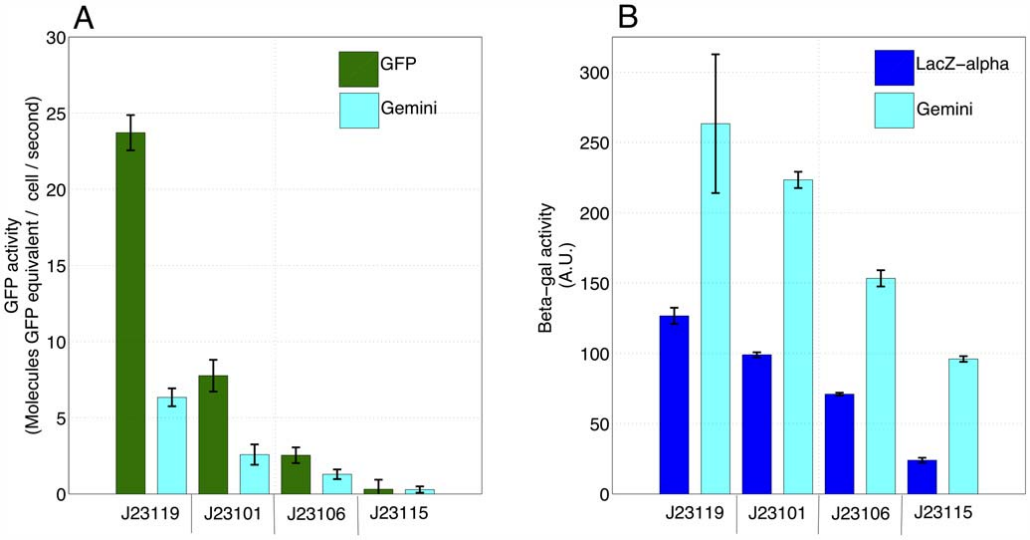
*Gemini* encodes stronger enzymatic activity than the β-gal α-fragment and weaker fluorescence activity than GFP. We wanted to quantitatively compare the fluorescence and enzymatic activities of *Gemini* to the individual activities of GFP and the β-gal α-fragment alone. **A**) We used a fluorimeter to measure arbitrary fluorescence (Figure S4) during a period of log phase growth for cells containing GFP and *Gemini* promoter variants. We averaged the fluorescence activity for each promoter variant (shown on the x-axis) across thirty minutes of mid-log phase growth for both GFP and *Gemini* (highlighted regions in Figure S5). We report the fluorescence activities for GFP and *Gemini* with error bars that represent the standard deviation of the averaged activity measurements. **B**) We also measured the enzymatic activity of *Gemini* relative to the β-gal α-fragment alone. We used a fluorogenic substrate for the β-galactosidase enzyme to quantify enzymatic activity in a Wallac Victor 3 fluorimeter (Methods). For the β-gal α-fragment and *Gemini* variants, we fit the fluorescence data with a linear regression. From this regression, we determined the enzymatic activity as the change in arbitrary fluorescence with respect to time. We calculated the average enzymatic activity for the β-gal and α-fragment *Gemini* across the three replicates for each promoter variant. We report enzymatic activities with error bars that represent the standard deviation of the averaged activity measurements.

Next, we more carefully measured the fluorescence activity of *Gemini* relative to GFP alone. We used a Wallac Victor 3 fluorimeter to measure absorbance (Figure S3) and fluorescence (Figure S4) during a period of log phase growth (Methods). For GFP promoter variants, we measured fluorescence activity as the change in GFP molecules per cell per unit time (Figure S5a). For *Gemini* promoter variants, we report the fluorescence activity as the change in molecules of GFP equivalent per cell per unit time (Figure S5b). We use molecules of GFP equivalent for two reasons. First, it allows direct comparison with the activity measured for GFP alone. Second, we do not have a calibration curve that allows us to calculate molecules of *Gemini* from arbitrary fluorescence data. We averaged fluorescence activity for each promoter variant across thirty minutes of mid-log phase growth for both GFP and *Gemini* (highlighted regions in Figure S5). We report fluorescence activities with error bars that represent the standard deviation of the averaged activity measurements (Figure 3a).

We measured the enzymatic activity of *Gemini* relative to β-gal α-fragment alone (Figure S6). We used a fluorogenic substrate for the β-galactosidase enzyme to quantify enzymatic activity in a Wallac Victor 3 fluorimeter (Methods). For the β-gal α-fragment and *Gemini* variants, we fit the fluorescence data with a linear regression. From this regression, we determined the enzymatic activity as the change in arbitrary fluorescence with respect to time (Figure S7 and Figure S8). We calculated the average enzymatic activity for β-gal and *Gemini* across the three replicates for each promoter variant (Figure 3b). We report enzymatic activities with error bars that represent the standard deviation of the averaged activity measurements.

**Figure 4:**
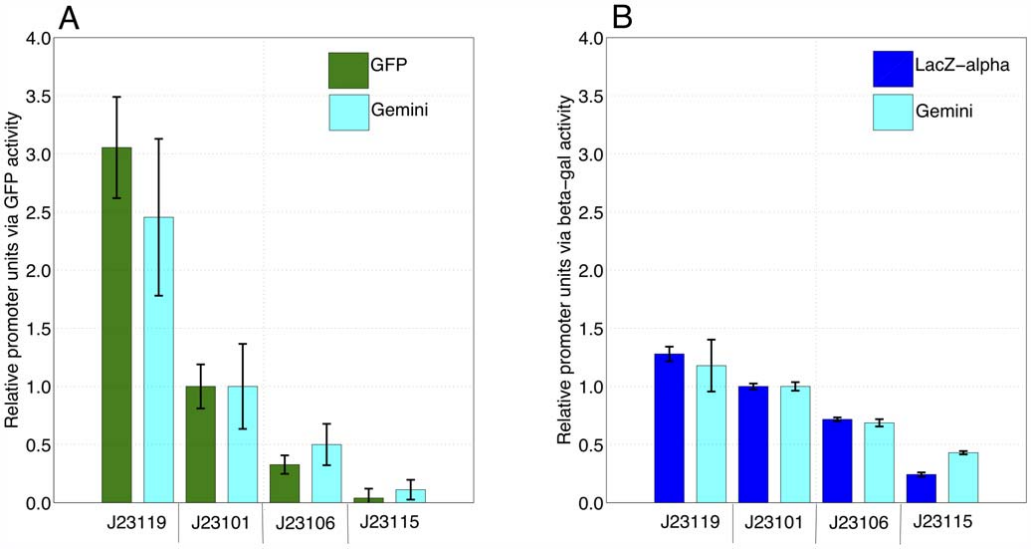
RPU measurements via *Gemini* are reasonably consistent with RPU measurements via GFP and the β-gal α-fragment. We measured the relative activity of each promoter via **A**) the fluorescence activity of GFP alone and *Gemini*, and **B**) via the enzymatic activity of the β-gal α-fragment alone and *Gemini*. We made RPU measurements by dividing the activity for each promoter (Figure 3) by the activity of promoter J23101 for the same reporter. We report promoter activities in relative promoter units (RPU) with error bars that represent the standard deviation of the relative activity measurements.

We measured the relative activity of each promoter via the fluorescence activity of GFP alone and *Gemini*, and via the enzymatic activity of the β-gal α-fragment alone and *Gemini*. We used promoter J23101 as a reference standard for all relative promoter measurements by dividing each measured activity (Figure 3) by the activity of promoter J23101 for the same reporter (Methods). We report relative promoter units (RPUs) with error bars that represent the standard deviation of the relative activity measurements (Figure 4).

## Discussion

### Design, construction, and characterization of *Gemini*, a bifunctional reporter

We describe the construction and quantitative characterization of *Gemini*, a fusion of the β-galactosidase (β-gal) α-fragment to the N-terminus of full-length green fluorescent protein (GFP) that is designed to support biological part characterization in microbes. We demonstrate that *Gemini* exhibits functional β-gal activity, which we assay with plates or fluorimetry, and GFP activity, which we assay with fluorimetry or microscopy. We show that complementation with the α-fragment fused to the N-terminus of full-length GFP is successful, and that *Gemini* exhibits more sensitive enzymatic activity than the β-gal α-fragment alone. We show that RPU measurements obtained via the fluorescence activity of *Gemini* are reasonably consistent with RPU measurements obtained via the fluorescence activity of GFP. We provide a calibration curve that allows engineers to relate RPU measurements obtained via the fluorescence activity of *Gemini* with RPU measurements obtained via enzymatic activity of *Gemini.*

### Functional composition of β-gal and GFP within *Gemini*

The enzymatic activity of *Gemini* is more sensitive than the activity of the β-gal α-fragment alone and the fluorescence activity of *Gemini* is less sensitive than the activity of GFP alone. This observation may be explained by the relative stabilities of GFP and the β-gal α-fragment alone. GFP is a relatively stable reporter; for example, the reported *in vivo* half-life of GFP is ∼26 hours in cultured mouse cells [19]. The β-gal α-fragment is known to be a relatively unstable reporter; for example, the reported enzymatic activity of the α-fragment is ∼25% that of full-length β-galactosidase [20]. With these points in mind, the α-fragment may be stabilized by its fusion to GFP, explaining the greater sensitivity of the β-gal α-fragment in the context of *Gemini* relative to the β-gal α-fragment alone. Conversely, GFP may be relatively destabilized by fusion to the α-fragment. Regardless of the mechanism, *Gemini* exhibits a wider dynamic range than either GFP or the α-fragment alone. Because it is more sensitive, the enzymatic activity of *Gemini* can be used to measure the activity of promoters that cannot be easily detected with the β-gal α-fragment alone (for example, Figure 2a, promoter J23115). Because the fluorescence activity of *Gemini* is weaker than that of GFP alone, *Gemini* may be used to measure stronger promoter activity than GFP alone; under the control of a strong promoter, GFP alone may saturate whereas the weaker fluorescence activity of *Gemini* may still be quantifiable (not shown).

### RPU measurements obtained via *Gemini*

Calculating the activity of a promoter that gives rise to a measured reporter protein synthesis rate requires a mathematical model. The parameter values within the model are sensitive to the experimental conditions, which often change from lab to lab and are generally difficult to measure. Thus, the transcriptional activity calculated for the same promoter often differs across laboratories [2]. Yet even if the parameter values governing reporter synthesis rate under a specific set of experimental conditions are unknown, the values may not change significantly between different cultures measured simultaneously within the same laboratory. The reporter synthesis rate calculated for a culture containing an uncharacterized promoter can be compared to the synthesis rate calculated for a culture containing a standard reference promoter grown in parallel. Ideally, the resulting ratio captures only the relative transcriptional activity between the uncharacterized promoter and the reference promoter, since all relevant parameters, except the promoter itself, are consistent between the two experiments. With this in mind, relative promoter units (RPUs) were developed to improve consistency in promoter activity measurements across laboratories and conditions. Using such as approach, a common reference promoter (J23101), and GFP, one study reduced the reported variability in measurements of promoter activity across laboratories by 50% [2].

Ideally, the RPU framework could be extended to reporter proteins besides GFP, so that different laboratories could make consistent RPU measurements with arbitrary reporters, and so that the synthetic biology community would only have to agree upon a common reference promoter. Clearly, parameter values such as maturation and degradation rates differ between reporter proteins. For example, there is a marked difference between the absolute fluorescence activity of GFP alone and *Gemini*, when both are expressed under the control of the same strong promoters J23119 and J23101, ceteris paribus (Figure 3a). Even if the parameter values that quantify the expression, maturation, and degradation rates of a reporter protein, such as *Gemini*, are unknown, the values may not change significantly when the reporter is placed under the control of different promoters. If these parameters do not change with respect to promoter activity, then RPU measurements obtained via *Gemini* should capture the relative transcriptional activity between the test and reference promoter, and should be consistent with measurements obtained via GFP alone.

RPU measurements obtained via the fluorescence activity of GFP are consistent within the bounds of measurement error with RPU measurements obtained via the fluorescence activity of *Gemini* (Figure 4a). This suggests that parameter values such as maturation and degradation rates for *Gemini* are reasonably insensitive to changes in promoter activity. If these parameters were sensitive to changes in promoter activity, a more significant divergence between RPU measurements obtained via GFP and *Gemini* would be expected. Instead, the results suggest that RPU measurements obtained via reporters other than GFP can be consistent with GFP, therefore extending the RPU framework to fluorescent reporters besides GFP.

However, we found an inconsistency between RPU measurements obtained via the fluorescence and enzymatic activities of *Gemini*. Specifically, we observed that at high expression levels the relative promoter measurements obtained via the fluorescence activity of *Gemini* (Figure 4a) diverge from the relative promoter measurements obtained via the enzymatic activity of *Gemini* (Figure 4b). One explanation for this divergence is that the enzymatic activity of *Gemini* may saturate if the quantity of expressed Gemini-encoded α-fragment exceeds the amount of complementing omega-fragment present within cells. Careful characterization of a functional full-length β-gal GFP fusion (unpublished results, Kortemme Lab, UCSF) would help to consider this model.

Finally, any future efforts to improve *Gemini* should note that Western blot experiments on cell extracts revealed two bands for *Gemini* (Figure S9). One band was near the size expected for *Gemini* (∼35.7 kDa) and the other appeared to be a cleavage product (∼30.7 kDa). Further Western blot experiments using a protocol optimized to improve size discrimination across this mass range yielded a band for the cleavage product at ∼30.1 kDa (Figure S9b). Given that the predicted mass for GFP plus the linker is ∼28.7 kDa, we expect that Gemini is being cut within the β-gal α-fragment (that is, ∼1.4–2.0 kDa beyond the linker). Thus, for *Gemini* and perhaps for any full-length β-gal GFP fusion, proteolytic cleavage may decouple the 1∶1 stoichiometric coupling of the two reporter epitopes. For now, and until perfect 1∶1 stoichiometric coupling can be demonstrated, RPU measurements obtained via the enzymatic activity of *Gemini* can be related to RPU measurements obtained via the fluorescence activity of *Gemini* using calibration curves (such as Figure S10).

## Materials and Methods

### Construction of reporter variants

We assembled Gemini via overlap PCR. We made constructs containing GFP (BBa_E0043) and LacZ-α (BBa_E0038) via separate PCR reactions. We performed one PCR reaction using LacZ-α as template with the primers one through three (below). We performed a second PCR using GFP as template with primers four through six (below). We gel purified each PCR product. We used a third PCR reaction to stitch the two products together using primers one and six to form the bifunctional reporter, Gemini (BBa_E0051). We Qiaquick gel purified the PCR product, digested it EcoRI and PstI restriction endonucleases, and cloned the digested PCR product into BioBrick vector, pSB1A3.

(1) tgccacctgacgtctaagaa

(2) gatgttcgctgccgccgccttcgctgccgcccgcgcgccattcgccattc

(3) cgctgccatgatgatgatgatgatgttcgctgccgccgccttc

(4) gaacatcatcatcatcatcatggcagcgaacgtaaaggagaagaacttttcac,

(5) gaaggcggcggcagcgaacatcatcatcatcatcatggcag

(6) attaccgcctttgagtgagc

We added RBS B0032 to *Gemini* via PCR. We performed PCR of the bifunctional reporter plasmid template using primers 6 and 7. We Qiaquick gel purified this PCR product, digested it with XbaI and PstI, and then performed a Qiagen ERC cleanup on the cut PCR product. We cut the destination vector pSB4A5 with EcoRI/PstI and then performed gel purification on the cut vector.

(7) gaattctctagagtcacacaggaaagtactagatgaccatgattacggattcac

We ligated different promoters to the digested PCR products containing the B0032 RBS and *Gemini*. We chose standard BioBrick promoters J23119, J23101, J23106, and J23115, because these promoters span a wide range of transcriptional activity and are commonly used by synthetic biologists. We synthesized both strands of each promoter variant as oligos containing an EcoRI overhang in front and SpeI overhang in the back. We normalized these oligos to 50 µM. For each oligo, we added 1 µl of the oligo to a 10 µl reaction with 1 µl 10x T4 DNA ligase buffer, 8 µl water, and 0.5 µl T4 PNK to phosphorylate the 5′ ends. After PNK treatment, we mixed the two complementary oligos together, heated them to 80°C, and allowed them to cool slowly. We ligated these promoters with the cut pSB4A5 vector and PCR products to form each of the promoter variants.

We wanted to fairly compare the fluorescence and enzymatic activities of *Gemini* to the individual activities of GFP and the β-gal α-fragment alone. To do this, we placed the coding sequences for GFP (BBa_E0043) and LacZ-α (BBa_E0038) under the control of the same expression cassettes, and on the same vector backbone, as *Gemini*. We used the Bricklayer assembly service provided by Ginkgo BioWorks to build the four GFP and four LacZ-α promoter variants, resulting in three sets of expression constructs: *Gemini* (Figure 1C), the β-gal α-fragment alone (Figure S2a), and GFP alone (Figure S2b).

### Measurement of fluorescence and enzymatic activity

We transformed the twelve expression constructs into *E. coli* TOP10 cells (Invitrogen). We grew the transformed *E. coli* on LB agar plates containing ampicilin (1∶1000 dilution) antibiotic. After overnight incubation, we inoculated 5 ml test tubes containing LB medium and ampicilin (1∶1000 dilution) with a single colony for each expression construct. After overnight incubation, we purified plasmid DNA for each expression construct using a mini-prep kit (Qiagen) and sequenced all expression constructs.

We used supplemented M9 medium (M9 salts, 1 mM thiamine hydrochloride, 0.2% casamino acids, 0.1 M MgSO4, 0.5 M CaCl2) with glycerol (0.4%) added as a carbon source and ampicilin (1∶1000 dilution) antibiotic for measurements of fluorescence activity for all expression constructs. We inoculated three test tubes containing 5 ml of sterile supplemented M9 medium with a single colony for each of the twelve reporter constructs. As a result, we grew three replicates for each of the twelve expression constructs. We grew the 36 cultures for approximately 18 hrs at 37°C. We then diluted each culture by a factor of 1∶100 into 5 ml of fresh media. We grew the diluted cultures for approximately five hours under the previous conditions.

After five hours, we measured the optical density of a 200 µl aliquot from each culture on a Wallac Victor3 multi-well fluorimeter (Perkin Elmer). We converted the optical density measurements from the fluorimeter to OD600 measurements using a standard calibration curve (not shown). We diluted the 36 cultures to the same optical density in 5 ml of fresh media and grew them for one hour at 37°C. We transferred a 200 µl aliquot from each of the 36 cultures into a well on a flat-bottomed 96 well plate (Nunc). We incubated the plate in a Wallac Victor3 multi-well fluorimeter at 37°C and assayed the samples with an automatically repeating protocol of absorbance measurements (600 nm absorbance filter, 0.1 second counting time), fluorescence measurements (485 nm excitation filter, 525 nm emission filter, 0.1 second measurement time), and shaking (20 second interval between measurements with 2.0 mm shaking diameter and double orbital shaking type).

We determined background absorbance by measuring wells containing only media, and background fluorescence by measuring the fluorescence of TOP10 cells without a GFP expressing vector. After background subtraction, we converted time-series fluorescence (Figure S4) and absorbance (Figure S3) measurements to GFP molecules per well and CFUs per well respectively, using standard calibration curves previously reported for the fluorimeter used in these experiments [1]. We calculated GFP synthesis rates (GFP molecules per cell per second) for all expression constructs containing GFP (Figure S5a). We also calculated GFP synthesis rates (molecules of GFP equivalent per cell per second) for all expression constructs containing *Gemini* (Figure S5b). We used molecules of GFP equivalent were used for two reasons. First, it allows direct comparison with the activity measured for GFP alone. Second, we do not have a standard calibration curve that allows us to calculate molecules of *Gemini* from arbitrary fluorescence data. We averaged the synthesis rates across thirty minutes of mid-log phase growth for both GFP and *Gemini* promoter variants (highlighted regions in Figure S5). We report the resulting fluorescence activities for GFP and *Gemini* with error bars that represent the standard deviation of the averaged activity measurements (Figure 3a).

After measurement of fluorescence activities, we measured enzymatic activity for each reporter in the three sets of expression constructs. We used *E. coli* TOP10 cells that contain the LacZ omega fragment necessary for LacZ-α complementation. We induced the LacZ omega fragment by growing cells in supplemented M9 medium with IPTG inducer (1∶1000 dilution). We used the flourogenic substrate 4-methylumbelliferyl-D-galactopyranoside (Sigma Aldrich #M1633), otherwise known as MUG, to measure β-gal activity. We dissolved the MUG in DMSO at a concentration of 2 mg/ml and used the resulting solution as a 10x stock. After measurement of fluorescence activities, we transferred a sample from each culture (10 µl) to a new well containing 20 µl of the MUG stock solution and PBS 170 µl for a total final volume of 200 µl. We were careful to ensure that cell density was consistent between the cultures prior to measuring β-gal activity (Figure S6). We measured fluorescence produced by reaction between β-gal and the MUG substrate using an automatically repeating protocol of measurements (355 nm excitation filter, 460 nm emission filter) on the Wallac Victor3 multi-well fluorimeter (Figure S7). We fit a linear regression to the time-series fluorescence data to measure the enzymatic activity for the β-gal α-fragment and *Gemini* expression constructs (Figure S8).

We measured the relative activity of each promoter via the fluorescence activity of GFP alone and *Gemini*, and via the enzymatic activity of the β-gal α-fragment alone and *Gemini*. We performed relative measurements using the method reported by Kelly et al [1]: we divided the measured activity (Figure 3) for each promoter by the activity of promoter J23101. We did this for the fluorescence activity of GFP alone, the fluorescence activity of *Gemini*, the enzymatic activity of the β-gal α-fragment alone, and the enzymatic activity of activity of *Gemini*. We report promoter activities in relative promoter units (RPU) with error bars that represent the standard deviation of the relative activity measurements (Figure 4).

## Supporting Information

Figure S1 Two designs for the bifunctional reporter of gene expression. A) In the first design, we fused the N-terminus of the β-gal α-fragment (BBa_E0038) to the C-terminus of full-length GFP (BBa_E0043). B) In the second design, we fused the N-terminus of full-length GFP to the C-terminus of the β-gal α-fragment with a linker.
(5.67 MB TIF)

Figure S2 Constructs used to benchmark the activity of *Gemini*. We wanted to fairly compare the fluorescence and enzymatic activities of *Gemini* to the individual activities of GFP and the β-gal α-fragment alone. To do this, we placed the coding sequences for each of the three reporters under the control of the same expression cassettes and vector backbones. This resulted in three sets of expression constructs. For each reporter, we built four promoter variants: *Gemini* (Figure 1C), A) β-gal α-fragment alone, and B) GFP alone.
(1.40 MB TIF)

Figure S3 Absorbance data and growth rates for GFP and *Gemini* promoter variants. We measured time-series absorbance for A) GFP and B) *Gemini* expression constructs on a fluorimeter and converted to OD600 using a calibration curve (Methods). We calculated growth rates for cells containing C) GFP and D) *Gemini* expression constructs for two reasons. First, growth rates were used to confirm that *Gemini* has no detrimental impact on cell growth relative to the stand-alone reporters. Two, growth rates were used to determine a regime of steady-state cell growth for subsequent measurements of reporter activity. Error bars represent the standard deviation of the averaged measurements for three replicates for each promoter variant.
(2.51 MB TIF)

Figure S4 Fluorescence and GFP concentrations for GFP and *Gemini* promoter variants. We measured time-series fluorescence for A) GFP and B) *Gemini* expression constructs with a fluorimeter (Methods). We calculated GFP concentrations for C) GFP D) *Gemini* promoter variants (Methods). Error bars represent the standard deviation of the averaged measurements for three replicates for each promoter variant.
(1.57 MB TIF)

Figure S5 Fluorescence activity for GFP and *Gemini* promoter variants. We calculated fluorescence activities for A) GFP and B) *Gemini* promoter variants using the fluorescence data shown in Figure S4 and the OD600 data shown in Figure S3 (Methods). We averaged fluorescence activities for thirty minutes of log phase growth, during which time was little fluctuation in the cell growth rate (Figure S3 c and d). Error bars represent the standard deviation of the averaged measurements for three replicates for each promoter variant.
(2.02 MB TIF)

Figure S6 Optical density of cultures prior to enzymatic activity assay. We assayed enzymatic activities for promoter variants of the β-gal α-fragment and *Gemini* at a consistent starting cell density.
(1.81 MB TIF)

Figure S7 MUG fluorescence for β-gal α-fragment and *Gemini* promoter variants. We measured time-series fluorescence produced by reaction between the MUG substrate and β-gal for A) the β-gal α-fragment and B) *Gemini* expression constructs with a fluorimeter (Methods). Error bars represent the standard deviation of the averaged measurements for three replicates for each promoter variant.
(1.99 MB TIF)

Figure S8 MUG fluorescence measured across replicates for each promoter variant of the β-gal α-fragment and *Gemini*. We measured MUG fluorescence for three replicates for each promoter variant of the β-gal α-fragment and *Gemini*. We fit a linear regression to the data for each replicate, and used the regression to calculate the enzymatic activity. We averaged enzymatic activities for the three replicates for each promoter variant of the β-gal α-fragment and *Gemini* (Figure 3b). We report enzymatic activities with error bars that represent the standard deviation of the averaged activity measurements.
(1.94 MB TIF)

Figure S9 Western blot for *Gemini* and GFP. A) We performed Western blot on cells containing *Gemini*, GFP, and the β-gal α-fragment expression constructs with promoter J23119 using anti-GFP antibody. Thus, the β-gal α-fragment is the negative control shown. Western blot reveals two bands for *Gemini*. One band is near the size expected for *Gemini*, and the other band appears to be a cleavage product of *Gemini*. B) We optimized Western blot protocol to achieve better discrimination between the sizes of the *Gemini* bands.
(1.23 MB TIF)

Figure S10 Calibration curve for *Gemini*. RPU measurements obtained via the enzymatic activity of *Gemini* can be related to RPU measurements obtained via fluorescence activity of *Gemini* using a calibration curve.
(0.83 MB TIF)
