# Supplementary figures and images for: Gemini, a Bifunctional Enzymatic and Fluorescent Reporter of Gene Expression
Source: PLoS One. 2009 Nov 4;4(11):e7569. doi: 10.1371/journal.pone.0007569 (PMC2766624; doi:10.1371/journal.pone.0007569)

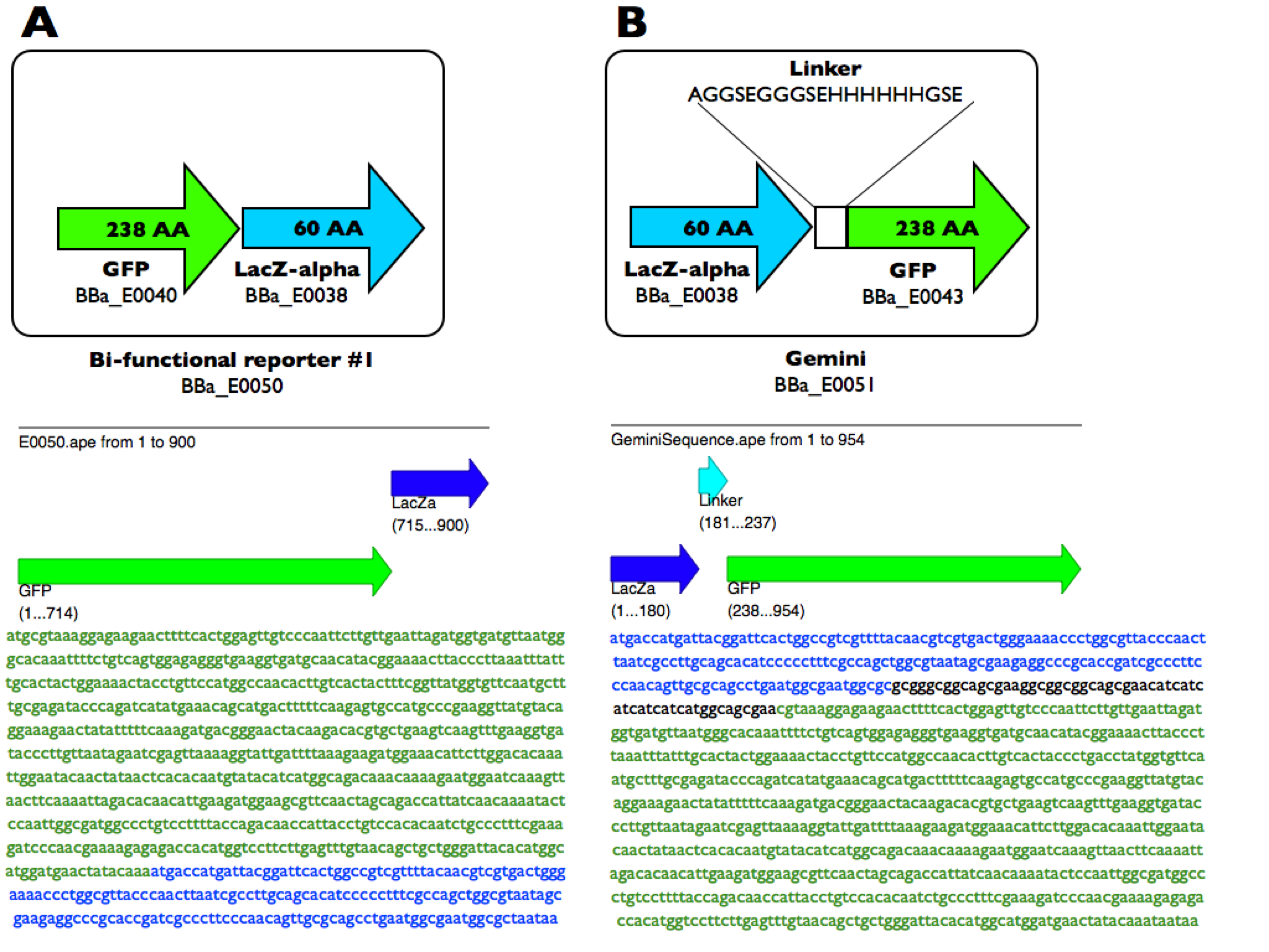

Supplement: Figure S1 — Two designs for the bifunctional reporter of gene expression. A) In the first design, we fused the N-terminus of the β-gal α-fragment (BBa_E0038) to the C-terminus of full-length GFP (BBa_E0043). B) In the second design, we fused the N-terminus of full-length GFP to the C-terminus of the β-gal α-fragment with a linker. (5.67 MB TIF) [file pone.0007569.s001.tif]

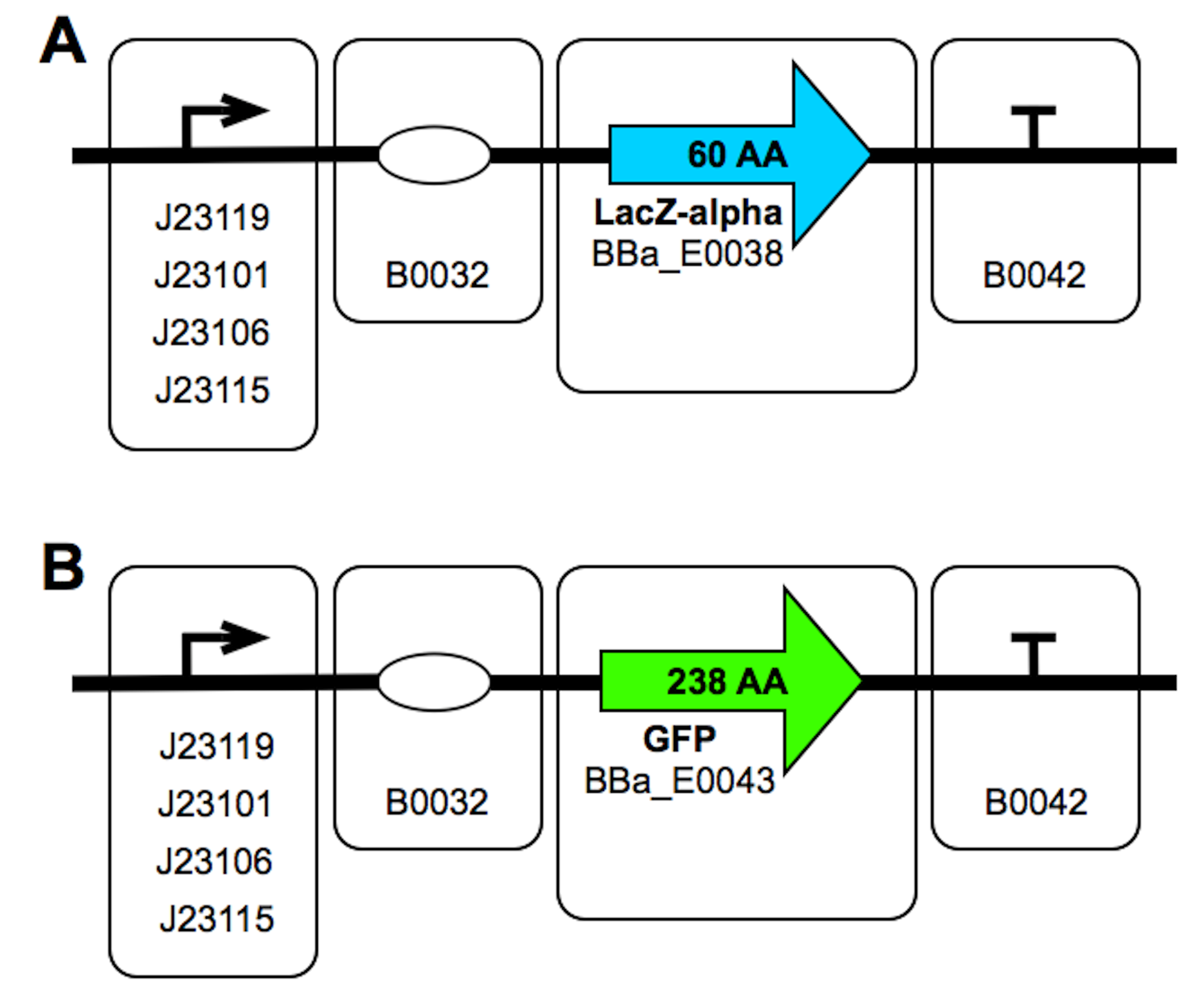

Supplement: Figure S2 — Constructs used to benchmark the activity of Gemini. We wanted to fairly compare the fluorescence and enzymatic activities of Gemini to the individual activities of GFP and the β-gal α-fragment alone. To do this, we placed the coding sequences for each of the three reporters under the control of the same expression cassettes and vector backbones. This resulted in three sets of expression constructs. For each reporter, we built four promoter variants: Gemini (Figure 1C), A) β-gal α-fragment alone, and B) GFP alone. (1.40 MB TIF) [file pone.0007569.s002.tif]

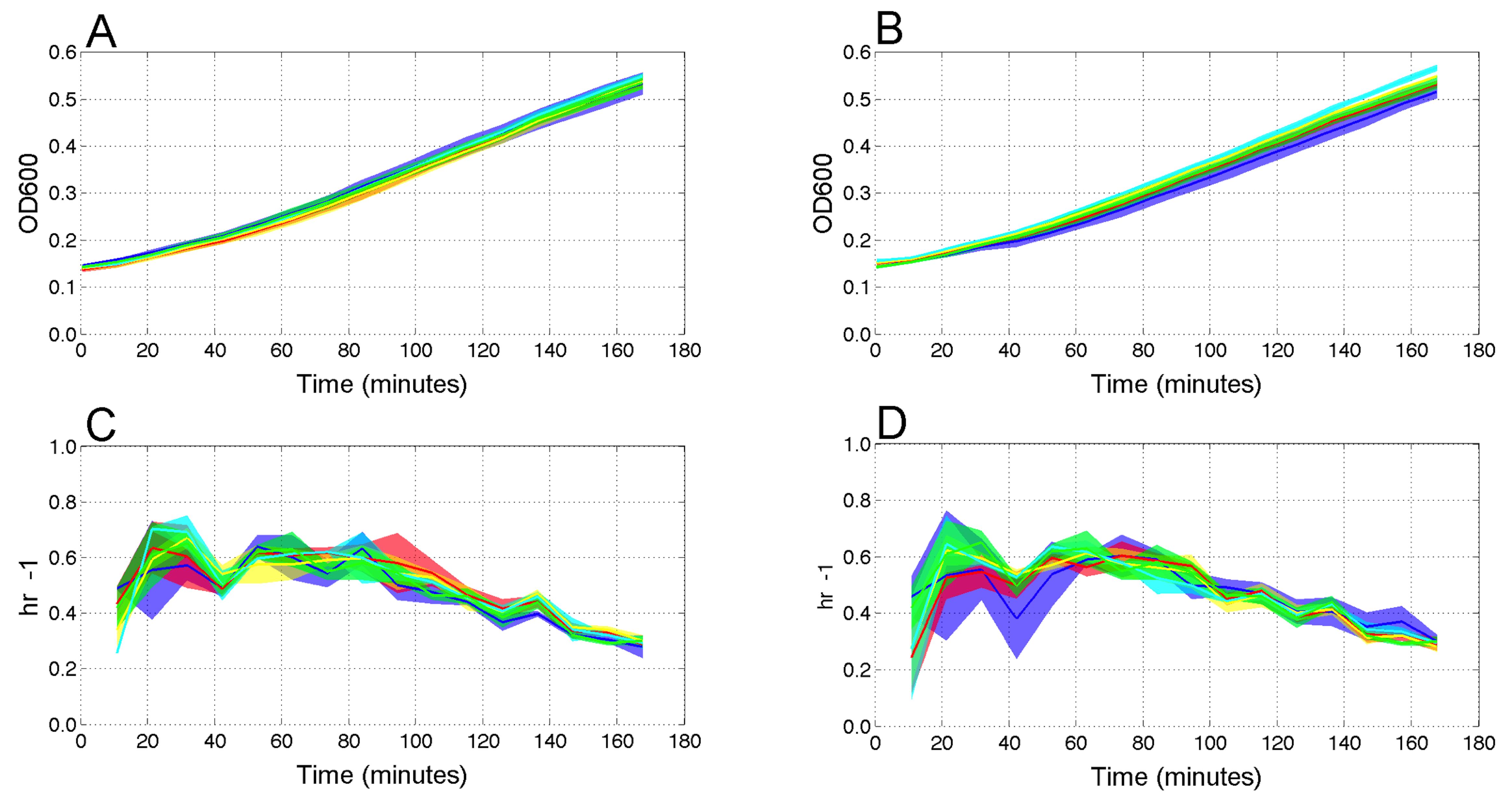

Supplement: Figure S3 — Absorbance data and growth rates for GFP and Gemini promoter variants. We measured time-series absorbance for A) GFP and B) Gemini expression constructs on a fluorimeter and converted to OD600 using a calibration curve (Methods). We calculated growth rates for cells containing C) GFP and D) Gemini expression constructs for two reasons. First, growth rates were used to confirm that Gemini has no detrimental impact on cell growth relative to the stand-alone reporters. Two, growth rates were used to determine a regime of steady-state cell growth for subsequent measurements of reporter activity. Error bars represent the standard deviation of the averaged measurements for three replicates for each promoter variant. (2.51 MB TIF) [file pone.0007569.s003.tif]

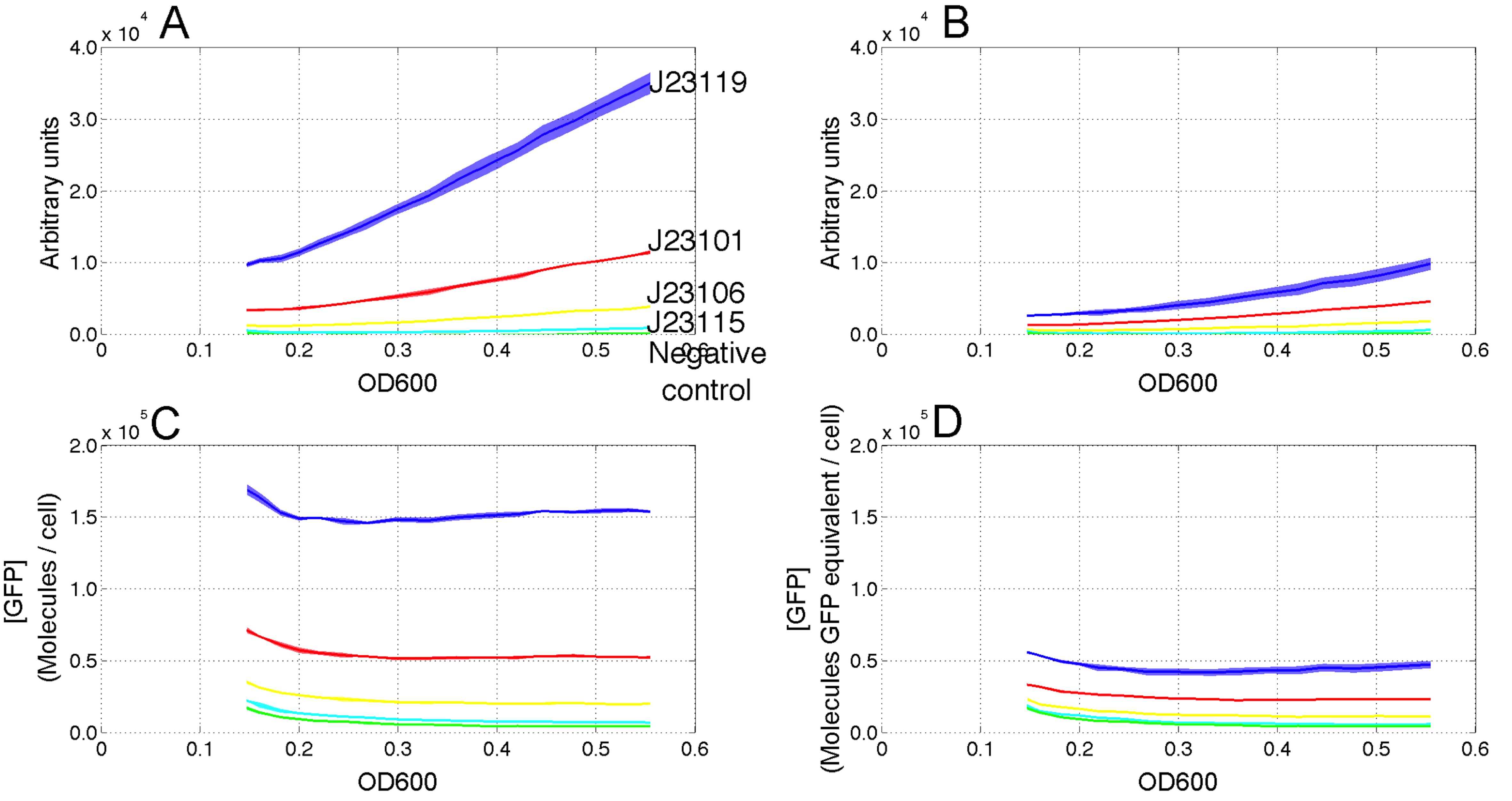

Supplement: Figure S4 — Fluorescence and GFP concentrations for GFP and Gemini promoter variants. We measured time-series fluorescence for A) GFP and B) Gemini expression constructs with a fluorimeter (Methods). We calculated GFP concentrations for C) GFP D) Gemini promoter variants (Methods). Error bars represent the standard deviation of the averaged measurements for three replicates for each promoter variant. (1.57 MB TIF) [file pone.0007569.s004.tif]

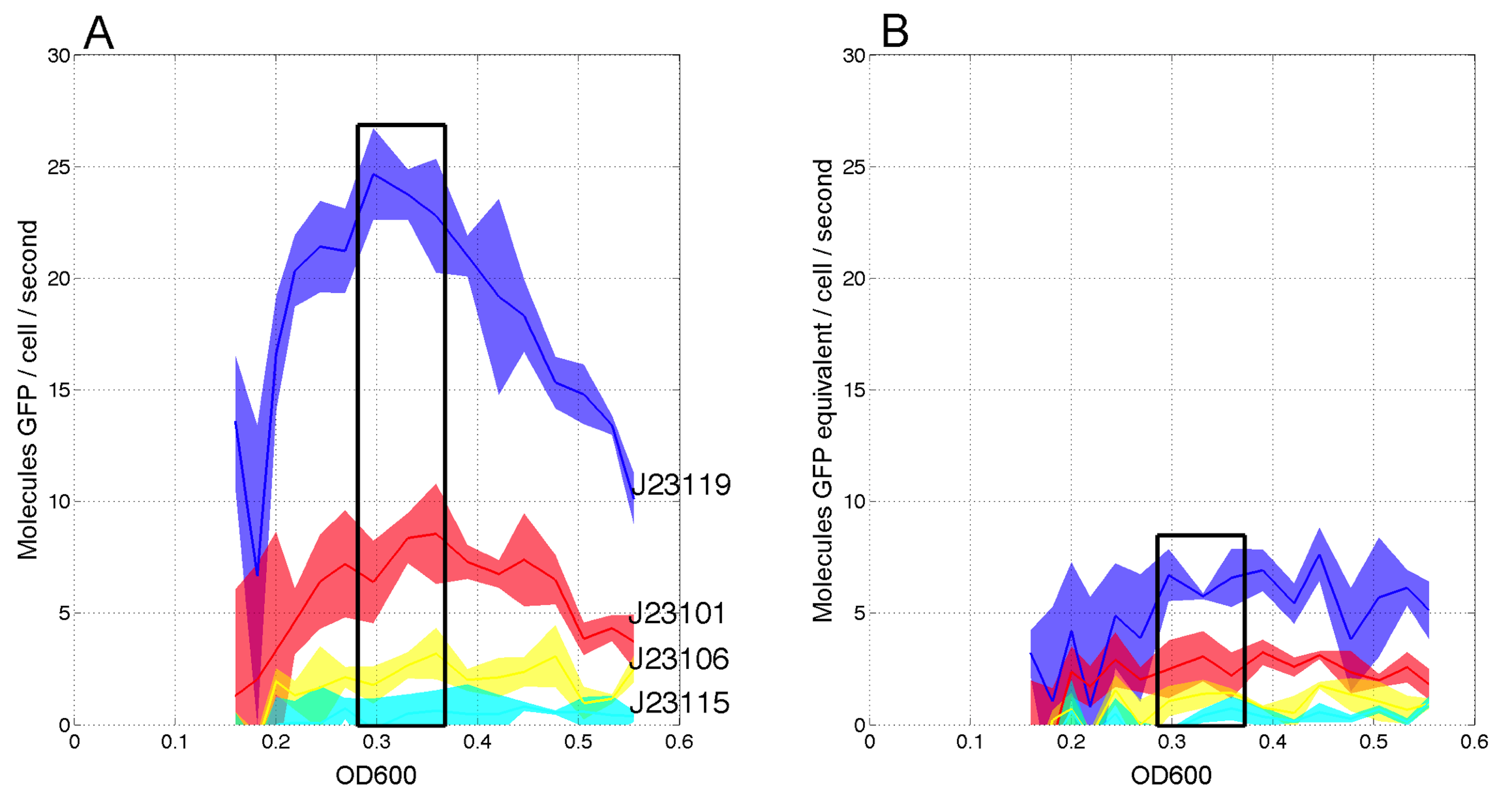

Supplement: Figure S5 — Fluorescence activity for GFP and Gemini promoter variants. We calculated fluorescence activities for A) GFP and B) Gemini promoter variants using the fluorescence data shown in Figure S4 and the OD600 data shown in Figure S3 (Methods). We averaged fluorescence activities for thirty minutes of log phase growth, during which time was little fluctuation in the cell growth rate (Figure S3 c and d). Error bars represent the standard deviation of the averaged measurements for three replicates for each promoter variant. (2.02 MB TIF) [file pone.0007569.s005.tif]

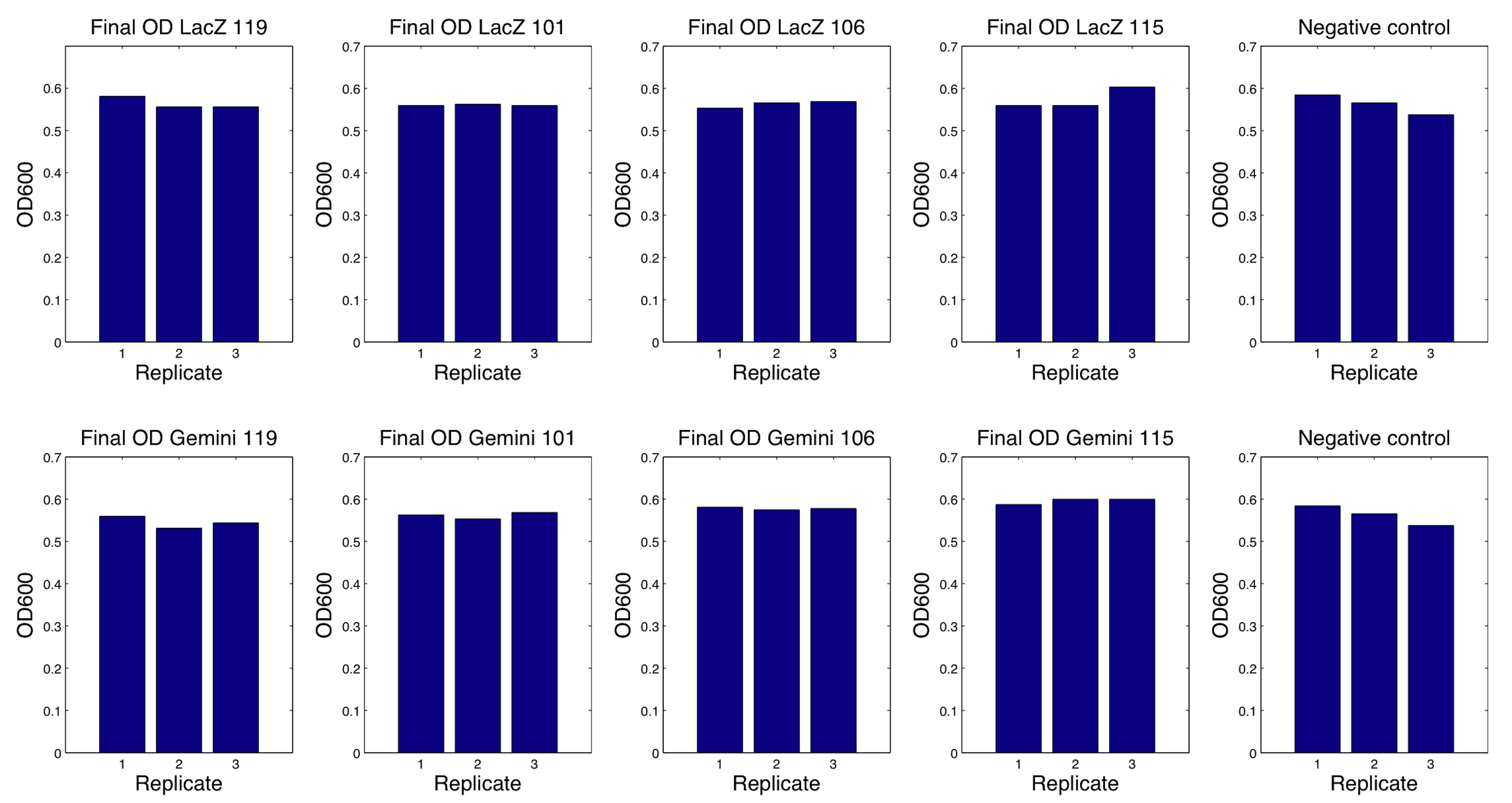

Supplement: Figure S6 — Optical density of cultures prior to enzymatic activity assay. We assayed enzymatic activities for promoter variants of the β-gal α-fragment and Gemini at a consistent starting cell density. (1.81 MB TIF) [file pone.0007569.s006.tif]

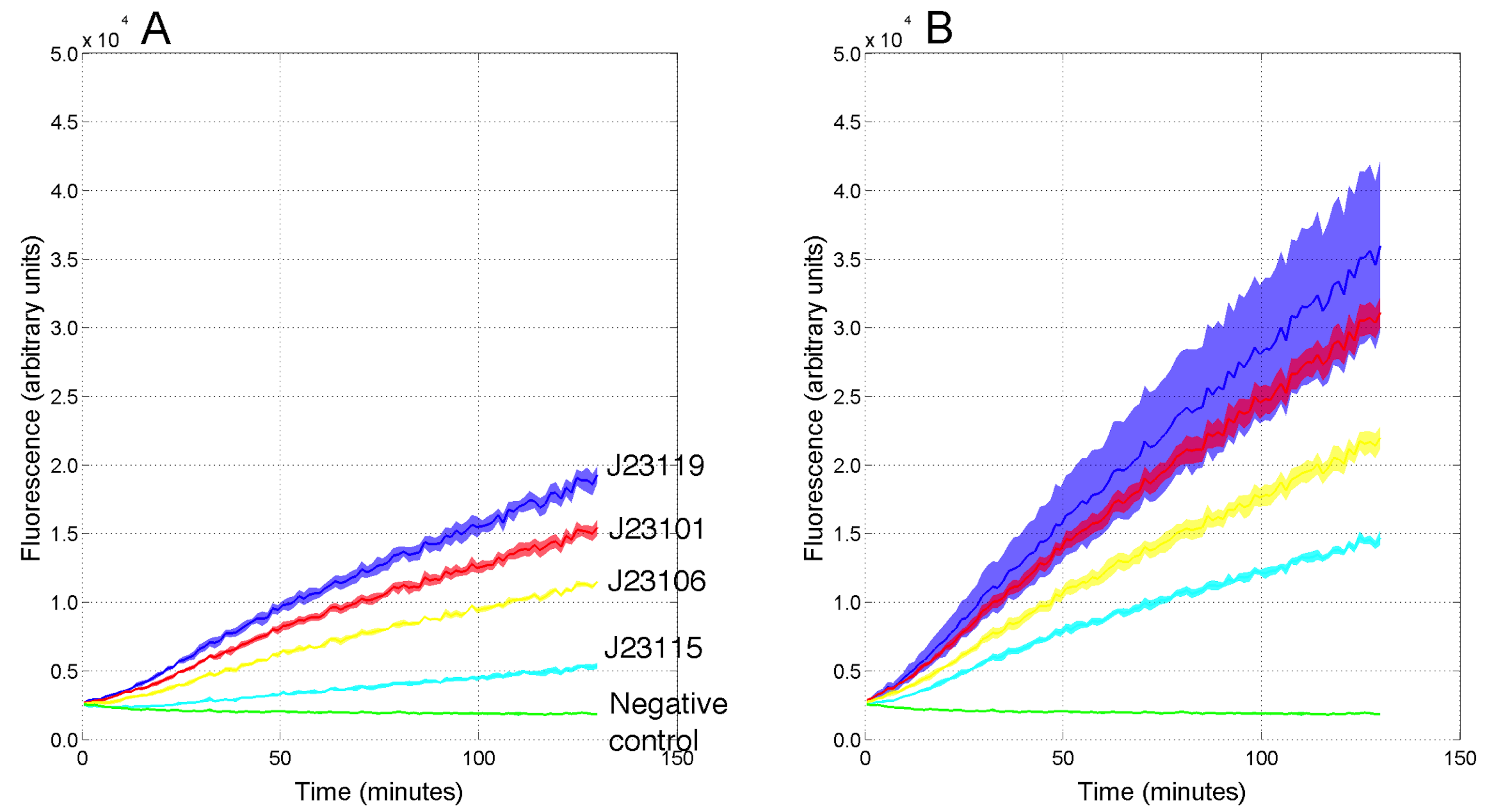

Supplement: Figure S7 — MUG fluorescence for β-gal α-fragment and Gemini promoter variants. We measured time-series fluorescence produced by reaction between the MUG substrate and β-gal for A) the β-gal α-fragment and B) Gemini expression constructs with a fluorimeter (Methods). Error bars represent the standard deviation of the averaged measurements for three replicates for each promoter variant. (1.99 MB TIF) [file pone.0007569.s007.tif]

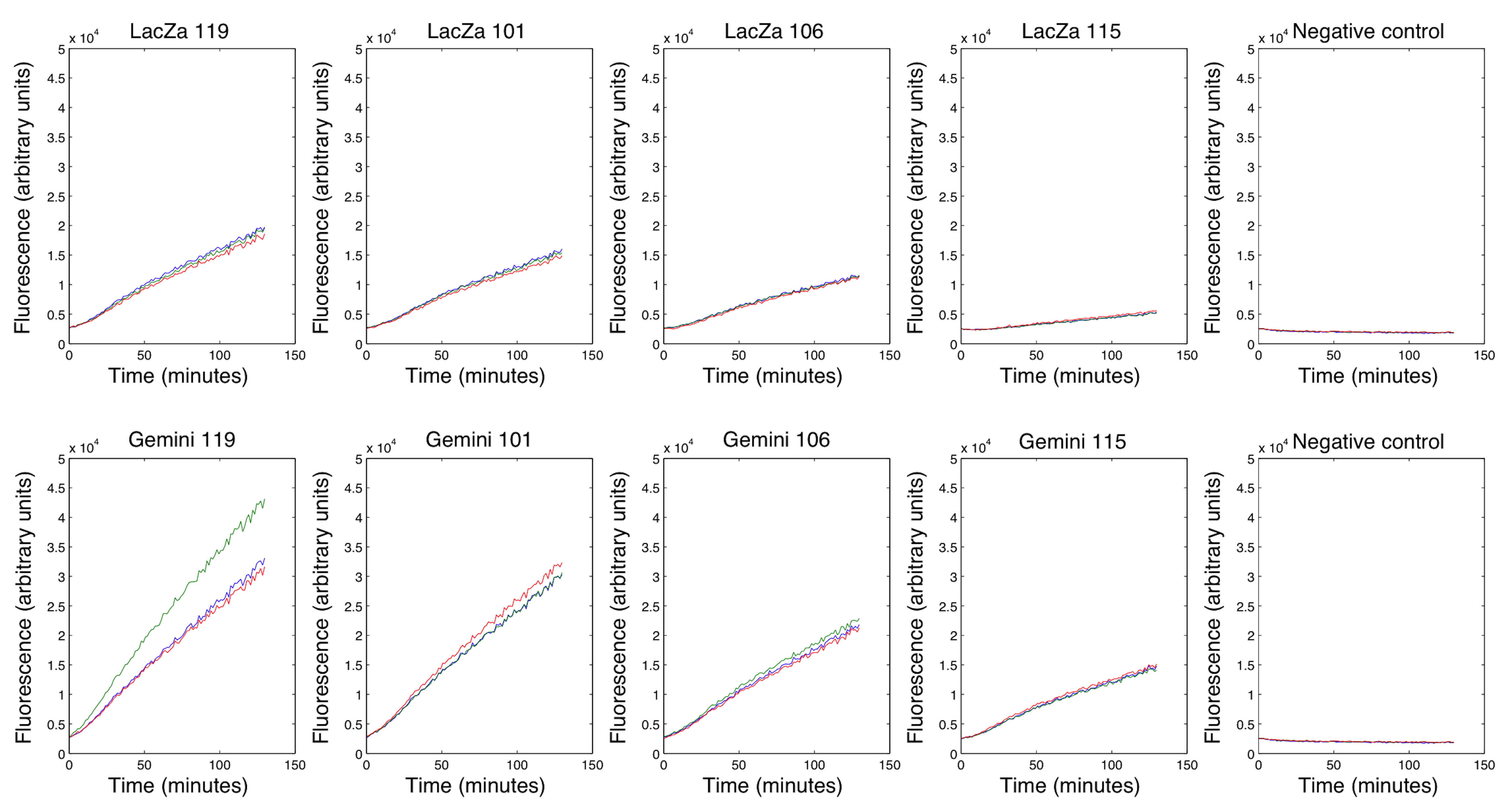

Supplement: Figure S8 — MUG fluorescence measured across replicates for each promoter variant of the β-gal α-fragment and Gemini. We measured MUG fluorescence for three replicates for each promoter variant of the β-gal α-fragment and Gemini. We fit a linear regression to the data for each replicate, and used the regression to calculate the enzymatic activity. We averaged enzymatic activities for the three replicates for each promoter variant of the β-gal α-fragment and Gemini (Figure 3b). We report enzymatic activities with error bars that represent the standard deviation of the averaged activity measurements. (1.94 MB TIF) [file pone.0007569.s008.tif]

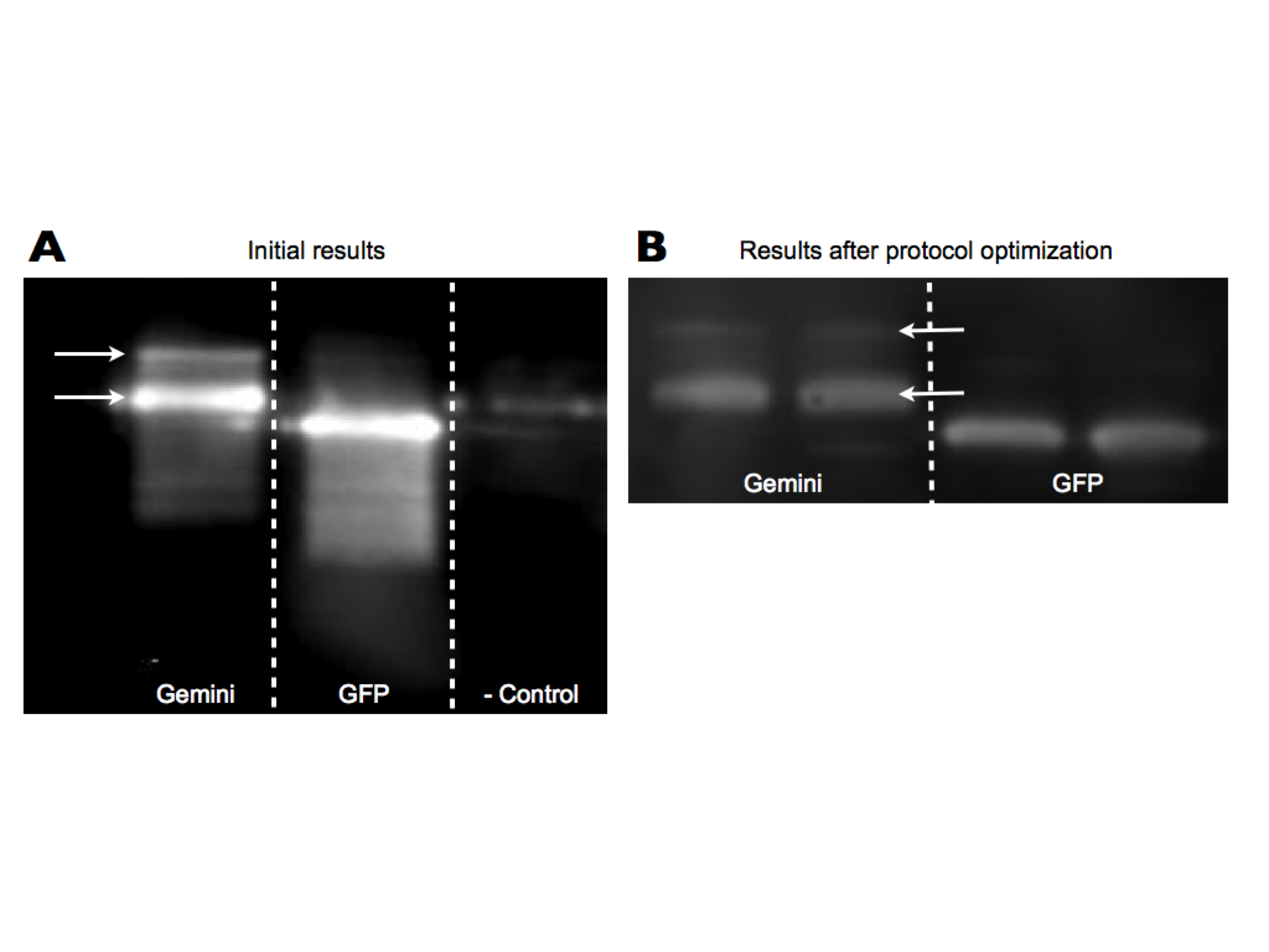

Supplement: Figure S9 — Western blot for Gemini and GFP. A) We performed Western blot on cells containing Gemini, GFP, and the β-gal α-fragment expression constructs with promoter J23119 using anti-GFP antibody. Thus, the β-gal α-fragment is the negative control shown. Western blot reveals two bands for Gemini. One band is near the size expected for Gemini, and the other band appears to be a cleavage product of Gemini. B) We optimized Western blot protocol to achieve better discrimination between the sizes of the Gemini bands. (1.23 MB TIF) [file pone.0007569.s009.tif]

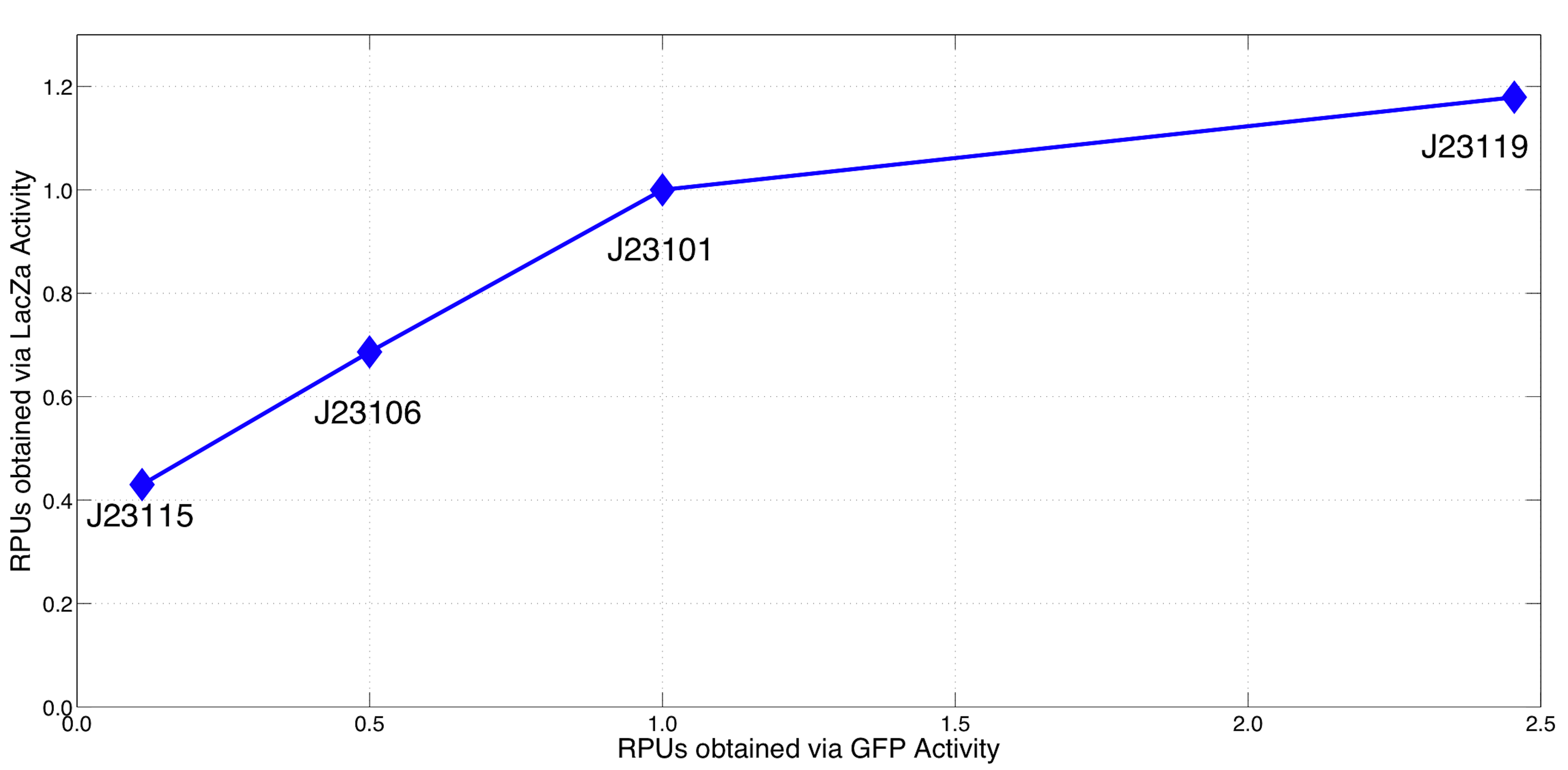

Supplement: Figure S10 — Calibration curve for Gemini. RPU measurements obtained via the enzymatic activity of Gemini can be related to RPU measurements obtained via fluorescence activity of Gemini using a calibration curve. (0.83 MB TIF) [file pone.0007569.s010.tif]
